# First Report of *bla*_VIM-4_- and *mcr-9*-Coharboring *Enterobacter* Species Isolated from a Pediatric Patient

**DOI:** 10.1128/mSphere.00629-19

**Published:** 2019-09-11

**Authors:** Kalyan D. Chavda, Lars F. Westblade, Michael J. Satlin, Andrew C. Hemmert, Mariana Castanheira, Stephen G. Jenkins, Liang Chen, Barry N. Kreiswirth

**Affiliations:** a Center for Discovery and Innovation, Hackensack Meridian Health, Nutley, New Jersey, USA; b Division of Infectious Diseases, Weill Cornell Medicine, New York, New York, USA; c Department of Pathology and Laboratory Medicine, Weill Cornell Medicine, New York, New York, USA; d BioFire Diagnostics, LLC, Salt Lake City, Utah, USA; e JMI Laboratories, North Liberty, Iowa, USA; Antimicrobial Development Specialists, LLC

**Keywords:** carbapenem, carbapenemase, colistin, *Enterobacter*, *mcr-9*, VIM, VIM-4

## Abstract

An Enterobacter hormaechei isolate harboring *bla*_VIM-4_ and *mcr-9* was recovered from a pediatric patient in a U.S. hospital. The *bla*_VIM-4_ and *mcr-9* genes are carried on the same IncH12 plasmid, pME-1a. The isolate tested susceptible to colistin, without observed induction of colistin resistance. The *mcr-9* gene is located between two insertion elements, IS*903* and IS*1*, but lacks the downstream regulatory genes (*qseC* and *qseB*) found in other isolates that harbor *mcr-9*.

**IMPORTANCE** We describe the complete genome assembly and sequence of a clinical *Enterobacter* isolate harboring both *bla*_VIM-4_ and *mcr-9* recovered from a pediatric patient in the United States with a history of travel to Egypt. Moreover, to the best of our knowledge, this is the first report of an *Enterobacter* isolate harboring both *bla*_VIM-4_ and *mcr-9* from the United States. The *bla*_VIM-4_ and *mcr-9* genes are carried on the same IncH12 plasmid, pME-1a. The isolate tested susceptible to colistin, without observed induction of colistin resistance. The *mcr-9* gene is located between two insertion elements, IS*903* and IS*1*, but lacks the downstream regulatory genes (*qseC* and *qseB*) found in other isolates that harbor *mcr-9*.

## Abstract (precis)

We describe the complete genome assembly and sequence of a clinical *Enterobacter* isolate harboring both *bla*_VIM-4_ and *mcr-9* recovered from a pediatric patient in the United States with a history of travel to Egypt. Moreover, to the best of our knowledge, this is the first report of an *Enterobacter* isolate harboring both *bla*_VIM-4_ and *mcr-9* from the United States. The *bla*_VIM-4_ and *mcr-9* genes are carried on the same IncH12 plasmid, pME-1a. The isolate tested susceptible to colistin, without observed induction of colistin resistance. The *mcr-9* gene is located between two insertion elements, IS*903* and IS*1*, but lacks the downstream regulatory genes (*qseC* and *qseB*) found in other isolates that harbor *mcr-9*.

## OBSERVATION

The rapid spread of carbapenemase-producing *Enterobacteriaceae* poses a significant global public health threat as therapeutic options are limited (1, 2). Carbapenemases are β-lactamases capable of hydrolyzing carbapenems and other β-lactams, leading to resistance to one of the most efficacious classes of antibiotics. These enzymes are grouped into molecular classes A, B, and D (3). Class B metallo-β-lactamases (MBLs) are capable of hydrolyzing penicillins, cephalosporins, and carbapenems but lack the ability to hydrolyze aztreonam (3). A member of this class, Verona integron-encoded MBL (VIM), has been reported in Pseudomonas aeruginosa, Acinetobacter baumannii, and members of the *Enterobacteriaceae* family (4). VIM-producing *Enterobacteriaceae* have been reported in several countries, especially in the Mediterranean region (4), including isolates from Kuwait (5) and the United Arab Emirates (4), but have rarely been described in the northeastern United States, where Klebsiella pneumoniae carbapenemase (KPC) is the predominate carbapenemase (1). As of 28 May 2019, 62 variants of *bla*_VIM_ have been reported in the National Database of Antibiotic-Resistant Organisms (NDARO) (https://www.ncbi.nlm.nih.gov/pathogens/antimicrobial-resistance/). Here, we report the comprehensive characterization of a clinical Enterobacter hormaechei isolate harboring *bla*_VIM-4_ from a New York City (NYC) patient using both the Illumina and Oxford Nanopore DNA sequencing platforms. Surprisingly, the genomic analysis identified the presence of the *mcr-9* gene on the same plasmid harboring *bla*_VIM-4_.

The *E. hormaechei* ME-1 isolate was recovered from a 3-year-old boy with the β-thalassemia trait who had a throat infection in Egypt 1 month prior to presentation at an NYC hospital in 2018. He was treated with an intramuscular injection of penicillin, but over the next 2 weeks he developed erythema and induration around the injection site. Two weeks later, he received 1 week of cephalexin, given the concern for cellulitis. He then presented to an emergency room in NYC with fever and a poorly healing wound with a central 1-cm eschar, and a surrounding rim of erythema was observed at the site of the prior intramuscular injection. He was found to have leukocytosis and an increasing percentage of bands on his blood count differential. A skin biopsy of the wound was performed. The Gram stain from the biopsy specimen demonstrated Gram-negative rods, and culture yielded an organism that was identified as a member of the Enterobacter cloacae complex by matrix-associated laser desorption ionization–time of flight mass spectrometry (MALDI Biotyper; Bruker Daltonics, Inc., Billerica, MA, USA), later identified as *E. hormaechei* by whole-genome sequencing (WGS). Using broth microdilution antibiotic susceptibility testing (6), the isolate tested resistant to ceftazidime, ceftriaxone, cefuroxime, doripenem, ertapenem, imipenem, meropenem, nitrofurantoin, piperacillin-tazobactam, ceftazidime-avibactam, ceftolozane-tazobactam, and trimethoprim-sulfamethoxazole; intermediate to tobramycin; and susceptible to amikacin, aztreonam, ciprofloxacin, doxycycline, levofloxacin, minocycline, tigecycline, tetracycline, and colistin. He was discharged prior to the availability of these culture results and was treated empirically with 2 weeks of oral clindamycin. The medical team was unable to reach his family after discharge to adjust his antimicrobial therapy. However, he was seen 3 months later, where his follow-up exam revealed that his rash had fully healed with a scar despite having never received treatment for *E. hormaechei*, suggesting that the organism was colonizing the wound but was not a pathogen.

The isolate was assayed on a research-use-only FilmArray antimicrobial resistance (AMR) panel (BioFire Diagnostics, LLC, Salt Lake City, UT, USA) utilizing the sample-to-answer BioFire system, and tested positive for both the *bla*_CTX-M_ and *bla*_VIM_ genes. To better understand the genetic structure of the *bla*_VIM_-harboring element and to confidently assemble all the plasmids contained in the strain, WGS was performed using a combination of the Illumina NextSeq (Illumina, San Diego, CA, USA) and MinION (Oxford Nanopore Technologies, Oxford, United Kingdom) platforms. Together, the sequence analysis generated high-quality assemblies and completely closed genomes of the isolate and all the plasmids harbored by the isolate.

Briefly, DNA was isolated from overnight cultures using a MasterPure Gram-positive DNA purification kit as recommended by the manufacturer (Epicentre, Madison, WI, USA). Libraries were prepared for sequencing using Illumina Nextera XT kits and sequenced on an Illumina NextSeq platform with paired 150-base sequence reads. In addition, a library for MinION sequencing was prepared using the Rapid Barcoding Sequencing kit (SQK-RBK004) and loaded onto an R9.4 flow cell. The run was performed on a MinION Mk1B device, and the sequencing reads were assembled using Unicycler (7).

The isolate contained a chromosome of 4,804,295 bp (Table 1) and 4 plasmids ranging in size from 2,317 bp to 276,502 bp. The average G+C content of the chromosome was 55.6%, and the chromosome harbored 5 prophages. The chromosome of ME-1 was compared to the genomes of the type strains of various *Enterobacter* species and found to belong to Enterobacter hormaechei subsp. *steigerwaltii* clade B Hoffmann VIII (8). *In silico* multilocus sequence typing (MLST) analysis assigned ME-1 to sequence type (ST) ST542 (allelic profile 178-4-4-6-37-4-6). *In silico* mining of antibiotic resistance genes revealed that the chromosome of ME-1 harbored *bla*_ACT-7_, an intrinsic AmpC enzyme, and *fosA*, which confer resistance to some β-lactams and fosfomycin, respectively.

**TABLE 1:** Key features of chromosome and plasmids harbored by ME-1

Sample name	Size (bp)	MLST	Plasmid incompatibility type	Antibiotic resistance gene(s)
Chromosome	4,804,295	ST542	NA^*b*^	*fosA*, *bla*_ACT-7_
pME-1a	276,520	NA	IncHI2	*aadA24*, *aadA2*,^*a*^ *sul1*,^*a*^ *dfrA1*, *dfrA16*,^*a*^ *mcr-9*, *bla*_VIM-4_, *bla*_CTX-M-9_, *qnrA1*
pME-1b	6,237	NA	ColRNAI	NA
pME-1c	2,496	NA	ColRNAI	NA
pME-1d	2,317	NA	ColRNAI	NA

a Multiple copies on the plasmid.

b NA, not applicable.

**FIG 1:**
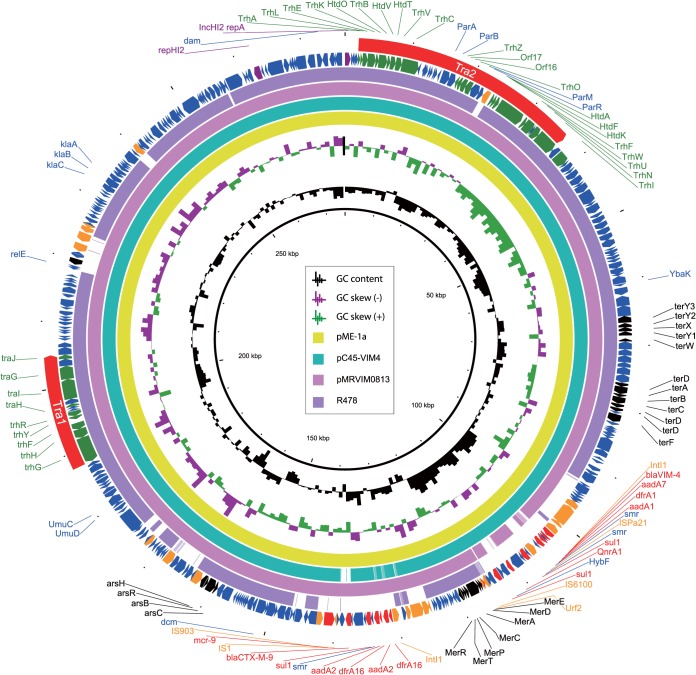
Plasmid structure of *bla*_VIM-4_- and *mcr-9*-harboring plasmid pME-1a compared to other IncH12 plasmids. Open reading frames are indicated by arrows and are colored based on predicted gene function: purple arrows indicate plasmid replication-associated genes, blue arrows indicate plasmid scaffold regions, green arrows indicate genes associated with conjugative transfer, orange arrows indicate accessory genes, and black arrows indicate heavy metal resistance genes. The regions for conjugative transfer, Tra1 and Tra2, are indicated by red arrows on the outer circle.

The isolate harbored a total of 4 plasmids (pME-1a, pME-1b, pME-1c, and pME-1d) belonging to incompatibility IncHI2 and ColE groups (Table 1). Analyses of acquired antibiotic resistance genes were performed using ResFinder 3.1 (9). Plasmid pME-1a harbored 12 antibiotic resistance genes conferring resistance to β-lactams (*bla*_VIM-4_ and *bla*_CTX-M-9_), aminoglycosides (*aadA2* and *aadA24*), sulfonamide (*sul1*), trimethoprim (*dfrA1* and *dfrA16*), and quinolones (*qnrA1*) (Fig. 1). Surprisingly, and with biological significance, a Blast analysis revealed that pME-1a also harbors the newly reported colistin resistance gene *mcr-9*, identical to the *mcr-9* gene identified in a *Salmonella* isolate (10). The other plasmids (pME-1b, pME-1c, and pME-1d) were not found to harbor any antibiotic resistance genes.

Plasmid pME-1a is 276,520 bp in length with an average G+C content of 46.3% and encodes 356 predicted open reading frames (Fig. 1). Full plasmid sequence queries of pME-1a against the NCBI GenBank (https://www.ncbi.nlm.nih.gov/genbank/) showed that it has high similarity to other IncH12 plasmids, e.g., pMRVIM0813 (98% query coverage and 99.99% sequence identity; GenBank accession number KP975077), pC45-VIM4 (98% query coverage and 99.98% sequence identity; GenBank accession number LT991958), and IncHI2 prototype plasmid R478 (79% query coverage and 99.97% sequence identity; GenBank accession number BX664015) (11). Similarly to plasmid R478, the conjugative transfer genes in pME-1a are also present in two separate regions, Tra1 and Tra2, presumably facilitating the dissemination of this plasmid between various members of the *Enterobacteriaceae* (Fig. 1) (11). Like plasmid R478, the Tra2 region in pME-1a encodes the majority of the mating pair formation (Mpf) complex, required for intracellular DNA transfer during bacterial conjugation, but there is an insertion of the IS*5* family transposase (IS*1182*) in the *parR* gene (11). The Tra1 region was similar to plasmid R478, harbored few Mpf complex genes, and encoded few OriT and relaxosome components (11).

The *bla*_VIM-4_ gene was identified on an In*416* integron, with the cassette structure of *bla*_VIM-4_-*aacA7*-*dfrA1*-*ΔssdA1*-*smr*-IS*Pa21*. The same integron has been found in an IncA/C plasmid containing *bla*_VIM-4_ in Klebsiella pneumoniae, Escherichia coli, and *Enterobacter* isolates from an outbreak in Kuwait (5). In*416* appears to be a common integron associated with the spread of *bla*_VIM_ (5). In addition, we found another novel integron with the cassette array of *dfrA16*-*aadA2*-*dfrA16*-*aadA2*-*smr*. Further examination of the plasmid sequences of pME-1a revealed the presence of a recently identified mobilized colistin resistance (*mcr*) gene, *mcr-9* (10). The gene *mcr-9* was initially recognized by *in silico* screening of sequenced Salmonella enterica subsp. *enterica* serotype Typhimurium (*S.* Typhimurium) genomes which revealed its sequence similarity to the *mcr-3* gene (10). The *S.* Typhimurium isolate harboring *mcr-9* had a colistin MIC value of 2 μg/ml, which is consistent with MICs observed for strains harboring other *mcr* alleles (12).

**FIG 2:**
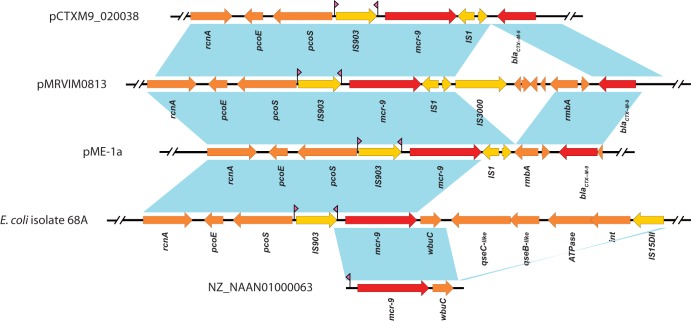
Comparison of the *mcr-9* region harbored by plasmids pCTXM9_020038 (GenBank accession number CP031724.1), pMRVIM0813 (GenBank accession number KP975077), and pME-1a (this study) and contigs from E. coli 68A (GenBank assembly GCA_900500325.1) and *S.* Typhimurium HUM_TYPH_WA_10_R9_3274 (NZ_NAAN01000063) (NCBI RefSeq accession number GCF_002091095.1). Blue shading denotes regions of shared homology among different plasmids and contigs. Colored arrows indicate open reading frames, with orange, yellow, and red arrows representing plasmid backbone genes, mobile elements, and antibiotic resistance genes, respectively. Purple arrowheads flanking IS*903* indicate the location of IS*903* inverted repeats.

In contrast, using the broth microdilution method according to Clinical and Laboratory Standards Institute guidelines (6), the isolate described here had a much lower colistin MIC value (0.12 μg/ml) (13). In pME-1a, *mcr-9* is located downstream of the *bla*_CTX-M-9_ gene (Fig. 1). Inspection of the regions surrounding pME-1 *mcr-9* reveal that it is located between two insertion elements, IS*903* (upstream) and IS*1* (downstream) (Fig. 2). Sequence query of the region surrounding *mcr-9* using NCBI GenBank showed similarity to other sequenced IncHI2 plasmids from *Enterobacter*: pCTXM9_020038 (GenBank accession number CP031724) and pMRVIM0813 (GenBank accession number KP975077) (Fig. 2). Analysis of the gene organization surrounding *mcr-9* in the *S.* Typhimurium HUM_TYPH_WA_10_R9_3274 isolate (10) revealed a unique cupin fold metalloprotein, WbuC, downstream of *mcr-9*, and interestingly, the upstream flanking region showed sequence homology to the inverted repeat region right (IRR) associated with IS*903* (GenBank accession number NZ_NAAN01000063) (Fig. 2). We are unable to determine whether a complete IS*903* element is upstream because the *mcr-9*-harboring plasmid associated with the *S.* Typhimurium isolate was not completely sequenced and only a short *mcr-9*-bearing contig (2,661 bp) is available for comparison (GenBank accession number NZ_NAAN01000063) (Fig. 2). This limitation highlights the importance of using long-read nanopore sequencing coupled with hybrid assembly to identify and monitor the transfer and rapid evolution of antibiotic resistance genes among bacteria.

A recent study from France reported a colistin-resistant isolate, E. coli 68A, harboring *mcr-9* on an IncH12 plasmid (14). Unlike *E. hormaechei* ME-1, the sequenced plasmid contig did not appear to harbor other antibiotic resistance genes (14). Interestingly, the analysis of the region surrounding *mcr-9* revealed the presence of insertion element IS*903* upstream of *mcr-9*, similar to the organization of pME-1a, pCTXM9_020038, and pMRVIM0813 (Fig. 2). However, unlike pME-1a, the *mcr-9*-harboring plasmid from E. coli 68A has a different downstream region bearing *wbuC*, *qseC*, *qseB*, and an ATPase gene (Fig. 2). In this study, the investigators were able to achieve clinical levels of colistin resistance by inducing the two-component system encoded by *qseB* and *qseC* using subinhibitory concentrations of colistin (14). In contrast, plasmids pME-1a, pCTXM9_020038, and pMRVIM0813 lack the *qseB* and *qseC* regulatory genes (Fig. 2). To further evaluate whether this two-component system is necessary for *mcr-9* induction in ME-1, we performed an induction assay and evaluated colistin resistance as described previously (14). Using subinhibitory concentrations of colistin at (0.03 and 0.06 μg/ml), the colistin MIC values of ME-1 did not change from 0.12 μg/ml, and gene expression of *mcr-9* was not elevated compared to expression in the absence of colistin (data not shown). The chromosomally carried *dnaJ* gene was used to normalize gene expression. These results confirm the importance of the *qseB*/*qseC* two-component system in the induction of colistin resistance mediated by *mcr-9*.

Consistent with the ability of *mcr-9* to confer colistin resistance, Carroll and colleagues placed *mcr-9* under the control of an exogenous and inducible promoter and demonstrated colistin resistance in E. coli NEB5α (10). Together, this finding suggests that the low-level expression of *mcr-9* in strain ME-1 is likely due to the absence of the *qseB*/*qseC* two-component regulators. However, we cannot rule out the possibility that the two insertion elements (IS*903* and IS*1*) that flank the gene in the pME-1a plasmid influence the expression of this resistance gene.

In summary, we describe the complete genome assembly and sequence of a clinical *Enterobacter* isolate harboring both *bla*_VIM-4_ and *mcr-9* recovered from a pediatric patient in the United States with a history of travel to Egypt. Moreover, to the best of our knowledge, this is the first report of an *Enterobacter* isolate harboring both *bla*_VIM-4_ and *mcr-9* from the United States. Studies are under way to better understand the role of the *mcr-9* gene and the contribution of the upstream IS*903* insertion in strain ME-1.

## 

### Accession number(s).

The complete nucleotide sequences of the chromosome of ME-1, pME-1a, pME-1b, pME-1c, and pME-1d were deposited as GenBank accession numbers CP041733 to CP041737, respectively.
